# Supplementary material for: LHCSR3-Type NPQ Prevents Photoinhibition and Slowed Growth under Fluctuating Light in Chlamydomonas reinhardtii
Source: Plants (Basel). 2020 Nov 18;9(11):1604. doi: 10.3390/plants9111604 (PMC7698959; doi:10.3390/plants9111604)
Supplement: Supplementary file 1 [file plants-09-01604-s001.pdf]

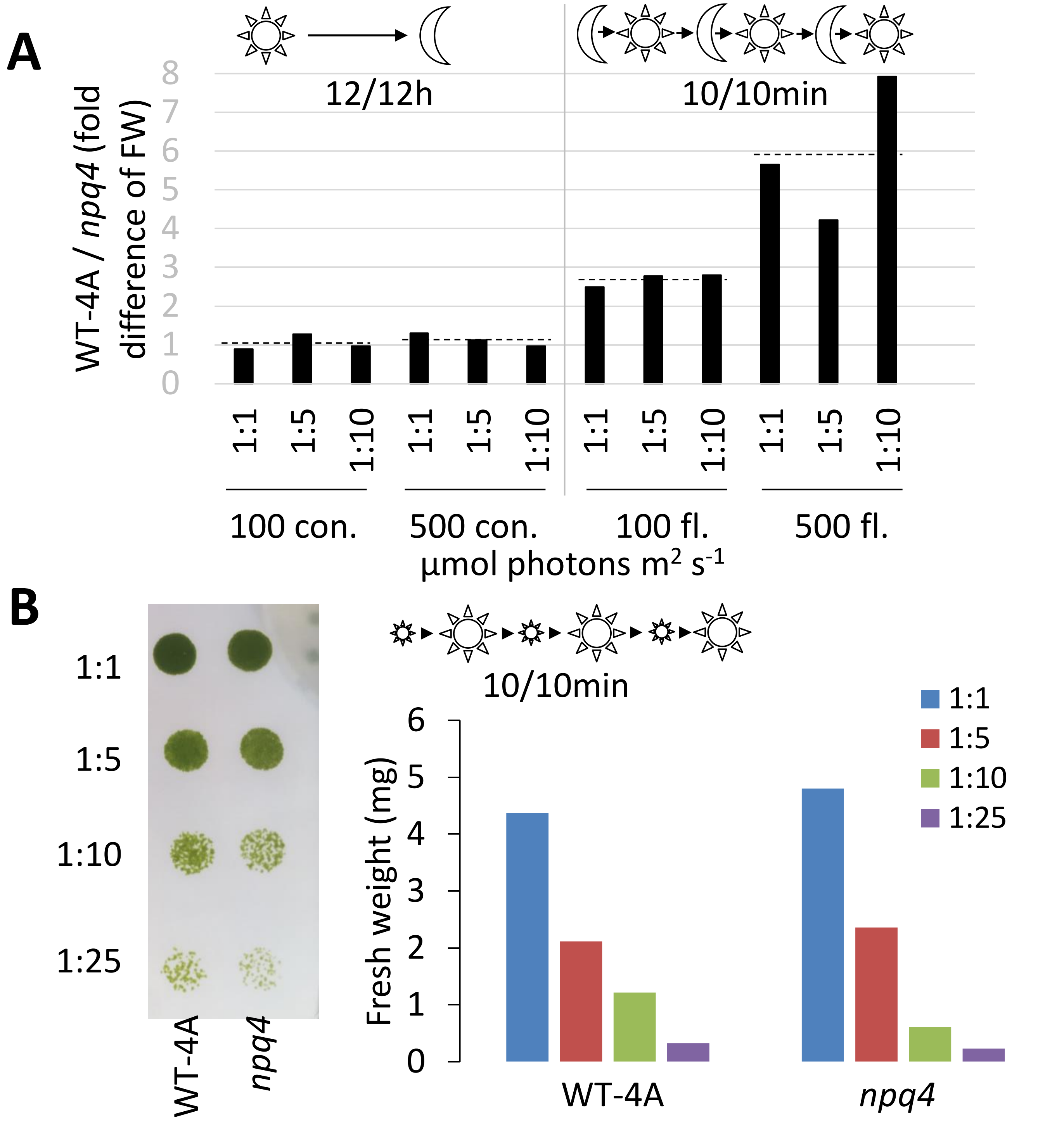

Supplementary Figure 1. Colony growth comparison of LHCSR3-deficeint *nqq4* mutant relative to wild type (WT-4A) under various light treatments. **(A)** Cells were cultured under repeated 10 minute fluctuations (fl.) of light intensity between 0 (moon) and 100 or 500 µmol photons m^{-2} s^{-1} (sun), or diurnal (12/12h) constant light (con.) at the same two intensities. Data is shown as fold difference between fresh weight of all WT-4A and *npq4* colonies at each initial culture dilution and after 6-8 days culturing (see methods). The average fold-difference is depicted by a dashed line. **(B)** Growth of *nqq4* and WT-4A under light fluctuating between 50 and 500 µmol photons m^{-2} s^{-1} (small sun, big sun). Representative images of colonies growing on agar are shown to the left of average colony fresh weight after 7 days of each initial culture dilution.

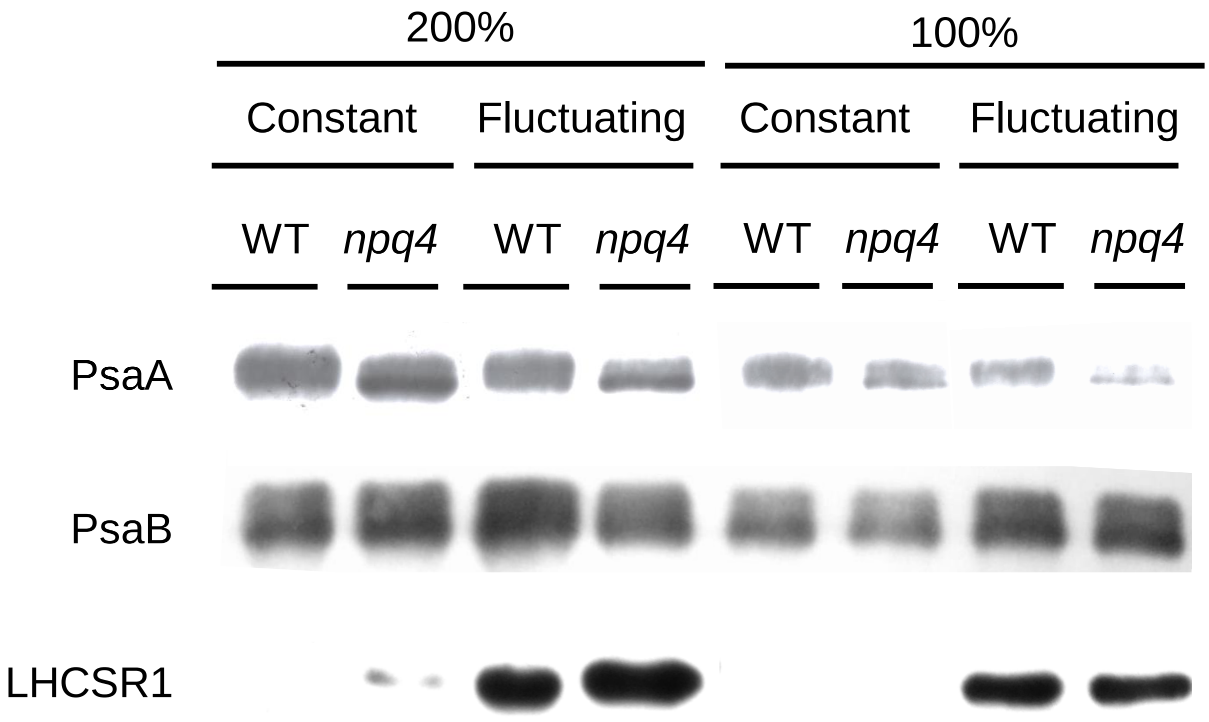

Supplementary Figure 2. Western blots of proteins from WT and *npq4* cells under constant or fluctuating light at 500µmol photons m^{-2} s^{-1}. For PsaA, proteins were loaded at 20µg (200%) or 10µg (100%) total protein, and for PsbA and LHCSR1, proteins were loaded at 2µg (200%) or 1µg (100%) total protein. All bands are from the same blot and transferred from the same gel.

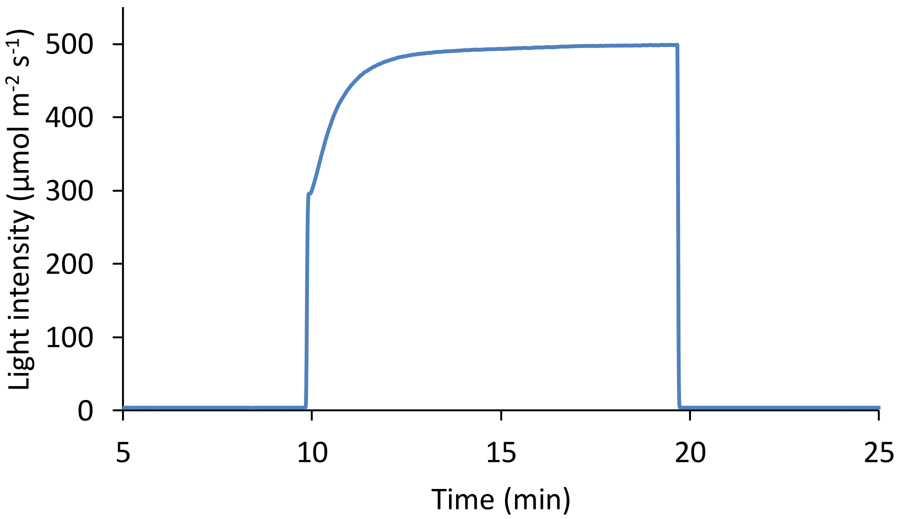

Supplementary Figure 3. The change in light intensity during 10min of fluctuating light, as measured with SQ-520 PAR sensor (Apogee Instruments).
